# Failure to Find a Conditioned Placebo Analgesic Response

**DOI:** 10.3389/fpsyg.2018.01198

**Published:** 2018-07-30

**Authors:** Magne A. Flaten, Espen Bjørkedal, Peter S. Lyby, Yngve Figenschau, Per M. Aslaksen

**Affiliations:** ^1^Department of Psychology, Norwegian University of Science and Technology, Trondheim, Norway; ^2^Department of Psychology, University of Tromsø, Tromsø, Norway; ^3^Department of Medical Biochemistry, University of Tromsø, Tromsø, Norway; ^4^Department of Medical Biochemistry, University Hospital of North Norway, Tromsø, Norway

**Keywords:** placebo effect, pain, classical conditioning, sex, gender

## Abstract

**Background:** Associative learning has, in several studies, been modulated by the sex of the participant. Consistent with this, a recent review found that conditioned nocebo effects are stronger in females than in males.

**Purpose:** It has been suggested that conditioned placebo responses are stronger in females, and this hypothesis was investigated in the present study. Cortisol and measures of negative emotions were taken to investigate if these processes could mediate any conditioned placebo effects.

**Methods:** Cold pain was applied to the volar forearm. The Conditioned group received inert capsules prior to two presentations of less painful stimulations, to associate intake of the capsules with reduced pain. The pain control group received the same painful stimulation as the Conditioned group, but no capsules. The Capsule control group received the capsules in the same way as the Conditioned group, but no decrease in the painful stimulation. Participant sex was crossed across groups. It was hypothesized that in the Conditioned group, an expectation of reduced pain should be induced after administration of the capsules, and this should generate placebo analgesia, and mostly so in females.

**Results:** The Conditioned group reported lower pain during conditioning, and rated the capsules as more effective painkillers than the capsule control group. However, placebo analgesia was not reliably observed in the Conditioned group.

**Conclusion:** The placebo capsules were rated as effective painkillers, but this did not translate into a placebo analgesic effect. This could be due to violation of response expectancies, too few conditioning trials, and differences in pain ratings in the pre-test that could be due to previous experience with painkillers.

## Introduction

Placebo analgesia refers to a reduction in pain after administration of an inert treatment together with information that the proposed treatment will reduce pain (Flaten and Al’Absi, 2012). Placebo analgesia may be induced by a conditioning procedure, where administration of inert capsules are paired with a reduction in pain, and conditioning procedures often induce placebo effects that are stronger than those generated by verbal information alone (Forsberg et al., 2017). Conditioned placebo effects has been proposed to be stronger in females than in males (Klosterhalfen et al., 2009), and this was investigated in the present experiment.

There is some evidence of sex differences in placebo and nocebo responding, although most studies do not report on sex differences. A review (Vambheim and Flaten, 2017) of 18 studies across several response systems, found that placebo responses induced by verbal information were larger and more frequent in males. Conditioned nocebo responses, on the other hand, were stronger and more frequent in females. No studies where placebo responses were induced by classical conditioning had reported on sex differences. However, Klosterhalfen et al. (2009), who found larger conditioned nocebo responses in females, proposed that females also respond with greater placebo responses after a classical conditioning procedure. Klosterhalfen’s hypothesis fit with Dalla and Shors (2009), who put forth the hypothesis that there are sex differences in learning, including classical conditioning. A relatively large literature after the Dalla and Shors (2009) article has reported on sex differences in classical conditioning (Löwgren et al., 2017), and some have linked sex differences in learning to sex differences in the stress response (Merz and Wolf, 2017).

The aim of the present study was to investigate this in more detail. Specifically, it was investigated whether conditioned placebo analgesia was related to decreases in stress, that previously has been shown to be a mediator of placebo analgesia induced by verbal information (Lyby et al., 2012), and whether this could explain any sex differences in conditioned placebo analgesia. The present study tested this hypothesis by using a classical conditioning procedure to induce placebo analgesic responding. The classical conditioning procedure was modeled after studies that have used such procedures successfully (Voudouris et al., 1990; Benedetti et al., 2003; Watson et al., 2006; Klinger et al., 2007; Colloca et al., 2008), where administration of a placebo (the conditioned stimulus) has been paired with surreptitious reduction in the painfulness of a stimulus (the unconditioned stimulus). It is hypothesized that the pairing of the placebo with lower pain will induce an expectation that the placebo treatment is effective, and this expectation mediates the placebo analgesic response (e.g., Benedetti et al., 2003). Thus, there should be an association between the amount of reduction in pain (the unconditioned stimulus) and the magnitude of the conditioned placebo analgesic response. This was investigated in the present study by having participants rate how effective they found the placebo treatment to be, and correlating this rating with the placebo analgesic response.

Pain was induced by cooling of a thermode attached to the lower arm. Cold stimulation has been used successfully in previous research on placebo analgesia. Several studies, e.g., Charron et al. (2006) and Czerniak et al. (2016) have observed placebo analgesia using the cold pressor test.

In the present study, three groups received a pre-test with a painful cold stimulus to the arm for 2 min. Subsequently, the Conditioned group ingested a capsule containing corn starch before each of two applications of less painful stimuli to the arm, termed manipulation trials. The Capsule control group received the capsules, but no change in the pain stimulus in the manipulation trials, to control that the administration of the capsules alone did not affect pain. The Pain control group did not receive the capsules, but was exposed to the same pain stimulus intensities as the Conditioned group, to control that the reduction in the pain stimulus in the manipulation trials did not affect the results. Thus, this group controlled for the natural history of pain in this experiment. Placebo responding was tested in a post-test that was identical to the pre-test, except that the participants in the Conditioned and Capsule control groups ingested a capsule prior to the test. Ratings of how effective the capsules were in lowering pain were obtained. It was predicted that in the Conditioned group, an association should be formed between the capsule and the reduced pain, that should lead to reports of lower pain in the post-test, i.e., placebo analgesia. Stress was monitored by salivary cortisol and subjective report. It was investigated whether stronger conditioned placebo effects were observed in female participants, and whether this was modulated by stress.

## Materials and Methods

### Participants

Participants were recruited by announcements on the University of Tromsø campus and on the University Hospital, or after lectures. A total of 75 participants took part in the experiment. To make the study double-blind, six of the participants received acetaminophen instead of placebo so the experimenters could tell the participants that some of them would receive a painkiller. These six participants were removed from the analyses. Another six participants were removed from the analyses due to apparatus malfunction or experimenter error. Two participants were removed because they reported pain unpleasantness at one or lower during all measurements. The analyses were done on the 61 remaining participants (mean age 21.9 years, range 19–37 years, 34 females, 27 males), except for the cortisol analyses were one additional participant was lost due to apparatus failure. The participants had been instructed not to smoke or use nicotine- or caffeine-containing products on the day of the experiment, and to have at least 5 h of sleep the night before the experiment. All participants reported previous use of painkillers. All experimental sessions were run between 11.30 and 15.45 h. Exclusion criteria were previous serious disease or injury, including pain, allergy to any medication, or use of prescription medicine, except birth control pills. Pregnant women were not allowed to participate and all females were tested for pregnancy prior to inclusion in the study. Females were tested in the non-menses part of the menstrual cycle. The participants were informed that they would take part in an experiment where pain would be applied to the arm and that a painkiller would be tested. They were also informed that some of them would receive a placebo. All participants signed an informed consent form. The study was approved by the Regional Committee for Medical Research Ethics North-Norway (protocol 29/2007). Each participant received 200 NOK (about 25€) for their participation.

### Experimenters

Three female (30, 47, and 50 years of age) and two male experimenters (31 and 33 years of age) ran the experiments. Each experimental session was run by one experimenter. The females were research nurses at the University Hospital of North Norway, and the males were PhD students at the Department of Psychology. The experimenters were trained in the experimental procedures, and interaction with the participants was standardized.

### Pain Stimulation

A TSA II Neurosensory Analyzer (Medoc, Israel) with a 30 × 30 mm aluminum thermode was used to administer cold pain to the right volar forearm. Four pain stimulations of 2 min duration were applied to each participant, and the thermode was moved to different spots on the volar forearm between each stimulation, according to a predefined pattern.

### Subjective Pain, Stress, and Arousal Measurements

Pain intensity and unpleasantness were recorded by numerical rating scales (NRS) where the participant indicated vocally how intense and unpleasant the pain was on a scale from 0 (no pain), via 1 (pain threshold) to 10 (unbearable pain or the most unpleasant that could be imagined). The difference between pain intensity and pain unpleasantness was explained as in O’Neill and Parrott (1992). Stress and arousal were measured as in Price et al. (1983). The participants indicated vocally, on a scale from 0 to 10, their feelings on the dimensions tense–relaxed and nervous-calm that indexed stress, and energetic-tired and awake-sleepy that indicated arousal.

### Pain Threshold

Pain threshold was assessed by lowering the temperature in the thermode from the baseline of 32°C with about 1°C/s until the participant reported the stimulus to be painful. This procedure was performed four times with a 1 min pause between each threshold measurement. Pain threshold was defined as the mean of the three last stimulations.

### Effectiveness

Ratings of the effectiveness of the capsules were assessed by asking the participants to indicate vocally on a scale from 0 to 10 how well they felt the medication worked. Zero indicated no effect and 10 complete pain relief.

### Saliva Samples

Cortisol was measured in saliva samples that were taken before the lunch the participants had when arriving at the Department of Clinical Research, after the meal right before the start of the experiment, and after the end of the experimental procedures. Saliva samples were collected with cotton dental rolls, and stored in capped plastic vials in a refrigerator at about +2°C for a maximum of 3 days before being centrifuged at 4,000 rpm for 5 min. The saliva was pipetted and stored at −20°C until assay. Cortisol levels (nmol/L) were determined using radio immunoassay-coated tubes (Salivette; Sarstedt, Etten-Leur, the Netherlands).

### Procedure

Prior to the experimental procedure, all participants had been informed that they would take part in an experiment where an analgesic drug was being tested. The participants met at the Department of Clinical Research at the University Hospital of North Norway at least 1 h before the start of the experimental session. All participants were instructed to eat a light breakfast, and had lunch at the Department. Before lunch, the first saliva sample was taken, and the participant received a standardized lunch of two pieces of brown bread with butter and orange marmalade, and water to drink, between 10.45 and 12.45 h. All participants were run between 11.30 and 15.45 h. There was at least 1 h from the participant had finished lunch to the experimental procedure started. After lunch the participant filled in the Fear of Pain Questionnaire III and the Cattell 16 PF personality inventory, both in Norwegian translation.

About 1 h after lunch did the experimental procedure start. The participant was placed in a comfortable chair in a room at the Research Department. The room had the appearance of an ordinary room at a ward at the hospital. The participant was informed about the pain test and about how the participant was to report pain, stress, and arousal.

The participants were told that “this study investigates how different doses of a painkiller affects cold pain. The painkiller has been shown to effectively reduce other forms of pain, and is now being tested on cold pain. Some persons will receive the drug, others will not receive the drug.” The information was the same for all groups.

About 10 min afterward the second saliva sample was taken, and the thermode was placed on the right volar forearm and pain threshold was assessed. Thereafter, the temperature was lowered to −10°C for 2 min, and the participant reported pain intensity and pain unpleasantness, stress, and arousal after about 45 s and 1 min and 45 s after pain stimulus onset. This constituted the pre-test. After termination of the pain stimulus the thermode was removed from the arm of the participant.

Immediately after termination of the pre-test, the participants in the Conditioned and Capsule control groups received one transparent capsule containing a white powder. For six of the participants the capsule contained 75 mg acetaminophen. For the remaining participants the capsule contained 75 mg corn starch. The participants in the Pain control group did not receive a capsule.

Ten minutes after administration of the capsules, or at a similar point in time for the Pain control group, the first manipulation trial was performed. In the Conditioned and Pain control group temperatures were 0°C for 2 min. In the Capsule control group the temperature was −10°C. In the Conditioned group, increasing the temperature was hypothesized to generate an association between the capsule and reduced pain. The Pain control group had not received the capsule, and no association between the capsule and reduced pain should be generated. The Pain control group served to control that changes in the intensity of the pain stimulus did not cause any between-group differences in the post-test. The pain was kept constant in all tests in the Capsule control group, and the capsule should not be associated with reduced pain in this group.

Immediately after termination of the first manipulation trial, the participants in the Conditioned and Capsule control groups rated capsule effectiveness. Thereafter, the participants in the Conditioned and Capsule control groups received a second capsule, which was identical to the first capsule. The participants in the Pain control group did not receive a capsule.

Ten minutes after administration of the second capsule, or at a similar point in time for the Pain control group, the second manipulation trial was performed. In the Conditioned and Pain control group temperatures were +5°C for 2 min. In the Capsule control group the temperature was −10°C. In the Conditioned group, increasing the temperature even more compared to the first manipulation trial was hypothesized to strengthen the association between the capsule and reduced pain.

Immediately after termination of the second manipulation trial, the participants in the Conditioned and Capsule control groups rated capsule effectiveness. Thereafter, the participants in the Conditioned and Capsule control groups received a third capsule, which was identical to the first capsule. The participants in the Pain control group did not receive a capsule.

Ten minutes after administration of the third capsule, or at a similar point in time for the Pain control group, the post-test was performed. The post-test was identical to the pre-test; pain threshold was assessed firstly, then all participants received −10°C to the forearm for 2 min. Finally, the third saliva sample was taken. The total duration of the experimental procedure was about 1 h.

### Blinding

The participants were told that they could receive a painkiller or an inactive ingredient. The experimenters were told that some of the participants would receive a painkiller and some would receive an inactive ingredient. The experimenters were not informed how many of the participants that would receive an inactive ingredient. A total of six participants received 75 mg acetaminophen per capsule, and these participants were removed from the analyses. The capsules and the forms for scoring of pain, stress, and arousal were stored in one envelope, which was opened prior to onset of the experimental procedures. The experimenters had information on which group the participants were assigned to prior to onset of the experimental procedures, but did not have information on which participants that received active medication or placebo.

### Placebo Analgesia: Response Definition

The placebo analgesic response was assessed by analysing the changes in pain unpleasantness and pain threshold from the pre- to the post-test in the three groups. A placebo response would be indicated by an interaction of Group × Test.

### Design and Statistical Analysis

The design was a 3 Group (Conditioned, Capsule control, Pain control) × 2 Participant Sex × 2 Test (pre-test, post-test) × 2 Time (pain reported at 45 s, and 1 min 45 s after pain stimulus onset) mixed design, with the first two factors treated as between-subjects factors, and the last two factors treated as within-subjects factors.

The effects of the independent variables were analyzed by analysis of variance for repeated measures. Theoretically interesting effects were followed-up by contrast analyses. *Post-hoc* tests were performed by Tukey’s HSD test for unequal samples. All analyses were performed in Statistica 7.0 (Statsoft). Alpha levels were.05.

## Results

The pain unpleasantness and pain intensity data yielded generally similar results, and only the pain unpleasantness data are reported.

### Pre-test

**FIGURE 1:**
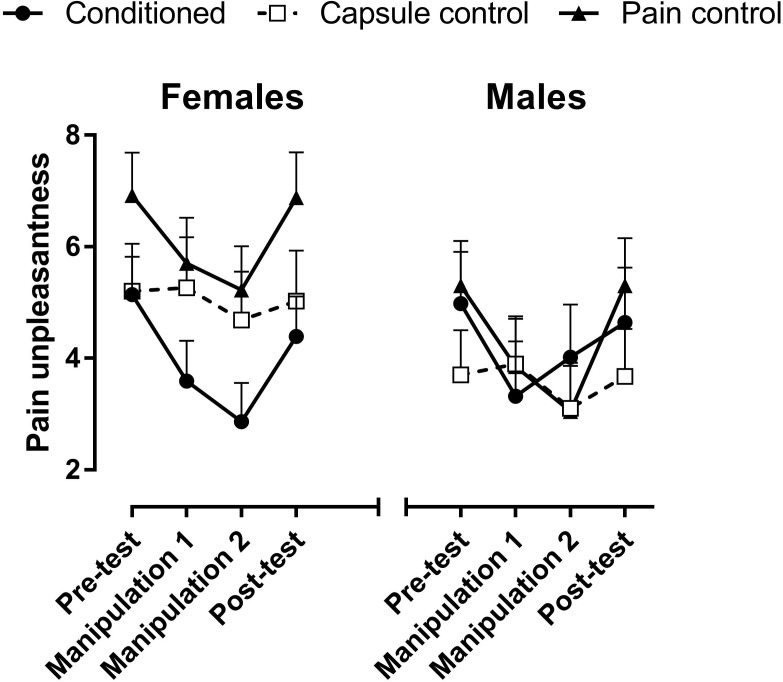
Mean pain unpleasantness across the four pain stimulation trials. In the Pre- and Post-tests, all groups received −10°C for 2 min to the forearm. The Conditioned group and Pain control group received 0°C and +5°C degrees in manipulation trials 1 and 2, respectively, whereas the Capsule control group received −10°C in all four trials. Error bars are +1 standard error of the mean.

Pain unpleasantness: Male participants reported slightly, but not significantly, less pain than female participants [*F*(1, 55) = 3.09, *p* = 0.084; *M* = 4.94 (SEM = 0.35) and 5.75 (0.31) for males and females, respectively; **Figure 1** and **Table 1**]. Pain increased across Time [from 45 s (*M* = 5.04 (SEM = 0.25)] to 1 min and 45 s [*M* = 5.68 (0.24) after application of the thermode; *F*(1, 55) = 12.64, *p* = 0.008, partial η^2^ = 0.22], but this increase was only observed in females and not in males, as evidenced by the interaction of Participant Sex × Time [*F*(1, 55) = 25.27, *p* = 0.000006, partial η^2^ = 0.45]. The main effect of Group [*F*(2, 55) = 2.66, *p* = 0.079] was not significant.

**Table 1:** Mean pain unpleasantness ratings across the four pain stimulation trials.

	Conditioned group		Capsule control group		Pain control group	
	Mean	SEM	Mean	SEM	Mean	SEM
**(A) Females**						
Pre-test	5,14	0,67	5,25	0,84	6,86	0,76
Manipulation1	3,58	0,68	5,25	0,85	5,63	0,76
Manipulation2	2,86	0,74	4,72	0,92	5,18	0,83
Post-test	4,39	0,77	5,02	0,96	6,90	0,87
**(B) Males**						
Pre-test	5,06	0,89	4,44	0,84	5,30	0,79
Manipulation1	3,43	0,90	4,72	0,85	3,85	0,80
Manipulation2	3,81	0,98	3,83	0,92	3,05	0,87
Post-test	4,78	1,02	4,36	0,96	5,30	0,91

*In the Pre- and Post-tests, all groups received −10°C for 2 min to the forearm. The Conditioned group and Pain control group received 0°C and +5°C degrees in manipulation trials 1 and 2, respectively, whereas the Capsule control group received −10°C in all four trials.*

Pain threshold: Males endured lower temperatures (had higher pain threshold) than females [*F*(1, 55) = 6.70, *p* = 0.013, partial η^2^ = 0.12; *M* = −6.8°C (SEM = 1.21) and −2.50°C (1.09), respectively]. No group differences were observed.

Stress: Males reported less stress, i.e., tension and nervousness than females [*F*(1, 55) = 12.39, *p* = 0. 0009, partial η^2^ = 0.22]. Stress increased slightly, but significantly, across Time [*F*(1, 55) = 3.71, *p* = 0.011, partial η^2^ = 0.12; *M* = 4.37 (SEM = 0.25) and 4.62 (0.25) at 45 s and 1 min 45 s, respectively].

Cortisol levels were similar across groups and participant sex in the pre-test.

No other main effects or interactions were significant in the pre-test data. See also Supplementary Materials.

### Manipulation Trials

Contrast analyses were performed to confirm that the manipulation trials decreased pain unpleasantness in the Conditioned and Pain control groups. Increasing the temperature from −10°C in the pre-test to 0°C in the first manipulation trial reduced pain unpleasantness ratings in the Conditioned group [*F*(1, 55) = 34.19, *p* = 0.000001] and the Pain control group [*F*(1, 55) = 24.95, *p* = 0.00006; **Figure 1**]. Increasing the temperature to +5°C in the second manipulation trial did not decrease pain further [*F* < 1 in the Conditioned group, *F*(1, 55) = 3.37, *p* = 0.08 in the Pain control group].

In the Capsule control group, where the temperature was kept at −10°C across the experiment, pain levels were stable across trials (pre-test vs. the first manipulation trial (*F* < 1, ns), and first vs. second manipulation trial [*F*(1, 55) = 3.69, *p* = 0.06].

### Placebo Effect

**FIGURE 2:**
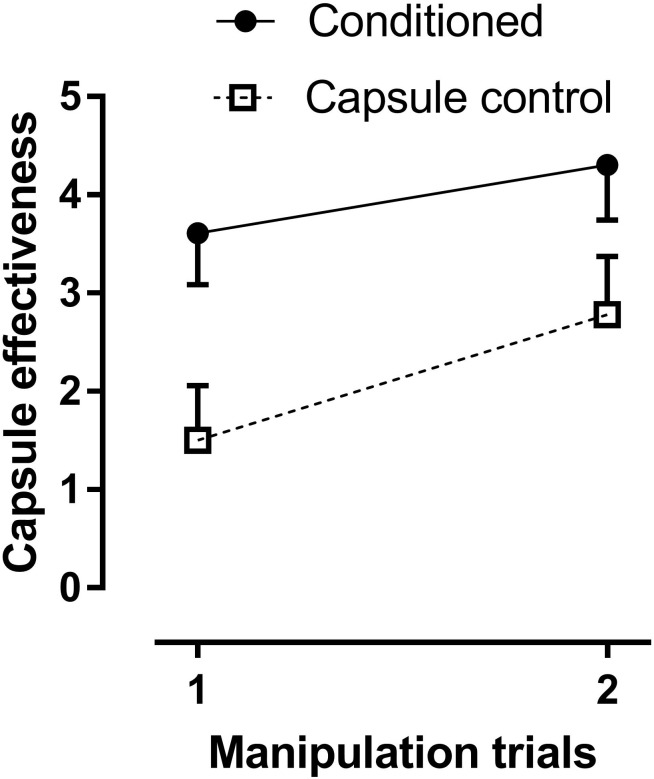
Means of rated capsule effectiveness in the two manipulation trials in the Conditioned group and the Capsule control group. Effectiveness ratings were significantly higher in the Conditioned group. However, note the increase in effectiveness ratings across trials also in the Capsule control group, even though the temperature was at −10°C in the pre-test and both manipulation trials in that group.

Capsule effectiveness: The Conditioned group rated the capsules as more effective painkillers than the Capsule control group [main effect of group: *F*(1, 36) = 6.27, *p* = 0.017, partial η^2^ = 0.18; *M* = 3.96 (SEM = 0.50) and 2.14 (0.53), respectively; **Figure 2**]. However, the effectiveness ratings in the Capsule control group were larger than 0, as evidenced by a *t*-test against 0 in the second manipulation trial [*t*(17) = 5.28, *p* = 0.00007]. Ratings of capsule effectiveness increased from the first to the second manipulation trial, as evidence by the significant main effect of Test [*F*(1, 36) = 9.55, *p* = 0.004, partial η^2^ = 0.27; *M* = 2.55 (SEM = 0.38) and 3.54 (0.41) for the first and second manipulation trial, respectively]. There was no main effect of sex on ratings of capsule effectiveness (p = 0.59).

As the experiment was run with two male and two female experimenters, who ran 31 and 30 participants, respectively, experimenter sex was included as a factor in the analyses of the capsule effectiveness ratings: The interaction of Group by Experimenter sex [*F*(1, 32) = 5.77, *p* = 0.023] was due to higher ratings in the Conditioned group only when a male acted as experimenter [Conditioned group = 5.01 (0.62) and Capsule control group = 1.75 (0.74)]. There was no difference between the groups when a female acted as experimenter [Conditioned group = 2.40 (0.73) and Capsule control group = 2.45 (0.67)]. The main effect of Experimenter sex was not significant [*F*(1, 32) = 1.92, *p* = 0.18] and no other interaction involving Experimenter sex was significant.

Rated capsule effectiveness correlated with the difference in pain report between the pre-test and the manipulation trials [*r*(39) = 0.49, *p* = 0.001, and *r*(39) = 0.43, *p* = 0.006, for the first and second manipulation trials, respectively].

Pain threshold: Males had higher pain threshold than females, as evidenced by a significant main effect of Participant sex [*F*(1, 55) = 6.15, *p* = 0.016, partial η^2^ = 0.11]. The interaction of Group × Test was not significant (*F* < 1, ns.).

Comparison of pain unpleasantness in the pre-test and the post-test: There was a main effect of Group [*F*(2, 55) = 4.01, *p* = 0.024, η^2^ = 0.15] due to higher pain unpleasantness in the Pain control group compared to the Conditioned group (*p* = 0.037) and the Capsule control group (*p* = 0.047). There was no interaction of Group by Test (*F* < 1, ns.) nor any other interaction involving these two factors.

As the conditioning manipulations were effective in reducing pain and increasing ratings of capsule effectiveness in the manipulation trials, it was investigated further why a significant placebo effect in the post-test was not observed in the Conditioned group. Thus, a mediation analysis was run in the Conditioned group with reduced pain in the second manipulation trial as the independent variable, effectiveness ratings in the second manipulation trial as the mediator, and the change in pain from the pre-test to the post-test as the dependent variable. The mediation analysis followed the recommendations from Baron and Kenny (1986) (**Table 2**): Firstly, the dependent variable was regressed on the independent variable: the reduction in pain in the second manipulation trial predicted reduced pain unpleasantness in the post-test at both 45 s and 1 min and 45 s after the onset of the painful stimulus. Secondly, the mediator was regressed on the independent variable, and the analysis showed that reduced pain at 45 s after the onset of the painful stimulus in the second manipulation trial also predicted increased ratings of capsule effectiveness. Reduced pain at 1 min and 45 s after stimulus onset did not predict capsule effectiveness, and was therefore removed from the last step in the analysis. Finally, the dependent variable of change in pain from the pre-test to the post-test was regressed on both the changed pain at 45 s after pain onset in the second manipulation trial and the second capsule effectiveness rating. The results showed that neither the reduced pain at 45 s after onset of the pain in the manipulation trials nor the effective rating predicted the change in pain from the pre-test to the post-test at any of the two measurements in the post-test.

**Table 2:** The steps in the mediation analysis investigating whether the change in pain from the pre-test to the post-test could be predicted by change in pain in the second manipulation trial, or by the second capsule effectiveness ratings.

Dependent variable	Predictor variable	*R*^2^	β	*p*
**STEP 1**				
Placebo analgesia 45 s	Manipulation trial 45 s	0.32	0.57	0.006
Placebo analgesia 1 min 45 s	Manipulation trial 1 min 45 s	0.34	0.58	0.005
**STEP 2**				
Capsule effectiveness	Manipulation trial 45 s	0.44	−0.66	0.0007
Capsule effectiveness	Manipulation trial 1 min 45 s	0.14	−0.37	0.11
**STEP 3**				
Placebo analgesia 45 s	Manipulation trial 45 s + capsule effectiveness	Whole model: 0.35		0.015
	Manipulation trial 45 s	0.32	0.41	0.12
	Capsule effectiveness	0.26	−0.23	0.26
Placebo analgesia 1 min 45 s	Manipulation trial 45 s + capsule effectiveness	Whole model: 0.20		0.11
	Manipulation trial 45 s	0.20	0.46	0.11
	Capsule effectiveness	0.08	0.02	

*Pain was recorded at 45 s and 1 min and 45 s after onset of each pain stimulus, and these recordings are analyzed separately. In the first two steps, separate regression analyses were run for each predictor. In the third analysis, both predictors were regressed on the dependent variable, as recommended by Baron and Kenny (1986).*

Comparison of stress in the pre-test and the post-test: There was a main effect of participant sex [*F*(1, 55) = 11.99, *p* = 0.001, partial η^2^ = 0.21] due to lower reported stress in male compared to female participants. The main effect of Test [*F*(1, 55) = 14.99, *p* = 0.0003, partial η^2^ = 0.27] was due to lower reported stress in the post-test compared to the pre-test. There was no effect of Group on stress, and no other main effects of interactions were significant in the stress data (all *F*s < 1.6, *p*s > 0.20).

Cortisol levels decreased slightly, but not significantly from the pre-test to the post-test [*F*(1, 54) = 3.12, *p* = 0.083, partial η^2^ = 0.05] but was not modulated by the experimental manipulations.

Comparison of arousal in the pre-test and the post-test: The main effect of Test [*F*(1, 50) = 56.52, *p* < 0.00001, partial η^2^ = 0.45] was due to lower reported arousal in the post-test compared to the pre-test.

No other main effects or interactions were significant.

## Discussion

The following were the main findings in the experiment: The conditioning procedure was successful in reducing pain unpleasantness. Furthermore, the reduction in pain unpleasantness during conditioning led to an increase in rated effectiveness of the capsules. Surprisingly, the placebo effect in the manipulation trials did not translate into a placebo effect in the post-test. Possible reasons for this will be discussed.

### Pre-test

Males had higher pain threshold and reported less pain unpleasantness than females. This is in accordance with a large literature on sex differences in pain (Greenspan et al., 2007; Fillingim et al., 2009) and will not be discussed further. Men also reported less stress than females, which also has been reported previously (e.g., Aslaksen et al., 2007).

### Manipulation Trials

Pain unpleasantness: Increasing the temperature from −10 to 0°C in the first manipulation trial decreased pain unpleasantness in the Conditioned group and the Pain control group. Thus, the manipulation trials were effective in reducing pain. Increasing the temperature to +5°C in the second manipulation trial did not decrease pain further, however. Pain ratings were stable across the manipulation trials in the Capsule control group where the stimulus was constant at −10°C across trials.

Capsule effectiveness ratings correlated with changes in reported pain during the two manipulation trials. Thus, the intake of the placebo capsules together with the reduction in pain, induced the belief that the capsules were effective in reducing pain, and that belief reduced reported pain in the manipulation trials. The capsules were rated as effective painkillers even in the Capsule control group, where pain levels were stable across the experiment. This is probably due to previous experience with painkilling capsules or tablets, and a placebo analgesic response could be expected also in the Capsule control group.

The pain unpleasantness data are similar to those obtained by Ruscheweyh et al. (2010), who applied a thermode with a temperature of +3°C to the back of the hand, and compared this to the cold pressor test with a temperature of +2.5°C. At 45 s after application (which is identical to the present study), the pain intensity reported to the thermode was about 4 on a scale from 0 to 10, while it was about 7.5 to pain induced by the cold pressor test. Thus, the cold pressure test induces much higher pain than the thermode, so the −10°C used in the pre- and post-tests in the present study cannot be compared to a similar temperature in the cold pressor test. Pain to the thermode increased from about 3.5 at 15 s to about 4 at 60 s, thus, only a slight increase in pain was observed across a period of 45 s. The data reported in the present study are quite similar. At 45 s after application of the thermode at 0°C (first manipulation trial) pain = 4.29 was reported. In the second manipulation trial +5°C in the thermode produced pain = 3.95. This compares well to the Ruscheweyh et al. (2010) who reported pain at about 4 to a +3°C thermode. Also, only a slight, but significant, increase in pain from 45 s to 1 min and 45 s was observed in the present study. No subject terminated the painful stimulation in the present study, whereas about 10% terminated the thermode test before 60 s in Ruscheweyh et al. (2010). This could be due to differences in procedures, as Ruscheweyh et al. (2010) presented a number of other painful stimuli, and used a different site for the application of pain.

### Placebo Effect

Capsule effectiveness ratings: the inert capsules were rated as effective even in the absence of an explicit conditioning procedure in the Capsule control group, as capsule effectiveness ratings were different from zero. Thus, it can be argued that intake of the capsules generated a belief that pain would be reduced also in the Capsule control group. However, the conditioning procedure had an effect in addition to the administration of the capsules alone had, as capsule effectiveness was rated higher in the Conditioned group compared to the Capsule control group. Thus, the placebo analgesic response should be larger in the Conditioned group, as capsule effectiveness was rated higher in this group. However, this belief did not translate into a placebo response in the post-test.

As the experiment was run with two male and two female experimenters, who ran 31 and 30 participants, respectively, experimenter sex was included as a factor in the analyses of the capsule effectiveness ratings. The capsule effectiveness ratings were higher when the capsules were administrated by a male compared to a female. Moreover, capsule effectiveness ratings were also higher in the Conditioned group compared to the capsule control group only when a male acted as experimenter. However, as there were a number of other differences between the male and female experimenters, conclusions about the reason for these findings cannot be drawn. The most notable differences were that the two males were PhD students in psychology, whereas the two females were nurses. The males were also younger than two of the females. These differences made it difficult to sort out the causes of any effects of experimenter sex.

Post-test: There was no Group by Test interaction to indicate a placebo response in pain threshold and pain unpleasantness. There are several reasons for this: firstly, a placebo response seems to has been elicited in the Capsule control group that was used to control for the effect of the capsules alone, i.e., control for the individual’s conditioning history involving pain medication. Thus, previous experience with pain medication seems to have elicited a placebo response, thus reducing the difference between the Conditioned group and the Capsule control group. Similar observations were made by Galer et al. (1997) and Levine and Gordon (1984), where subtle cues indicating that a painkiller had been administrated elicited a placebo analgesic response in the absence of explicit information that a painkiller had been administrated.

Secondly, although not significant, females in the Conditioned group and the Capsule control group rated pain in the pre-test about 1,7 points lower than females in the Pain control group. This difference would make it difficult to observe a placebo effect in females in the post-test. Again, this may be attributed to the knowledge of the experimenters of group belongingness, that could have affected experimenter behavior (Levine and Gordon, 1984; Gracely et al., 1985; Galer et al., 1997). A similar observation was made by Flaten et al. (2006) and we are at present investigating this issue.

Thirdly, the difference in temperatures between the manipulation trials and the post-test in the Conditioned group could have violated the expectations of pain relief. The present study was, however, modeled after previous successful studies on classical conditioning of placebo analgesia (Voudouris et al., 1990; Watson et al., 2006; Rhudy et al., 2018). In these studies, and in the present one, the stimulus in the post-test was identical to that in the pre-test. This procedure may have reduced placebo analgesia in the post-test, since the application of a more painful stimulus than expected in the post-test could have generated surprise due to the unexpectedly painful stimulus, and nervousness and arousal if the participant felt that something unexpected happened (Lyby et al., 2012). It has been reported (Mitchell et al., 1996) that a mismatch between an unexpected event, in that case not receiving a drug, and the actual event (d-amphetamine administration) generated increased anxiety. This could have been detrimental to the placebo analgesic effect.

Fourthly, the instructions in the present study told the participants that they may or may not receive a painkiller. Thus, a conditioning only procedure was used, and no verbal information was provided that could increase the placebo response in the Conditioned group compared to the Capsule control group. Other studies have used a similar procedure and found placebo analgesia (Voudouris et al., 1990; de Jong et al., 1996; Klinger et al., 2007), whereas a recent study (Rhudy et al., 2018) found no evidence of placebo analgesia in a conditioning only group. However, in Rhudy et al. (2018) and Voudouris et al. (1990) the participants were explicitly told that they would not receive a pain relieving cream. This information abolished any expectation of pain relief (Rhudy et al., 2018). Thus, there are inconsistencies in the literature regarding how a conditioning only procedure is performed, and the results from this type of procedure.

Several studies have used short visual cues that signal presentation of brief, painful stimuli of different intensities. Thus, one color is associated with less pain than other colors, but the participant is not informed about this association. When the different colors are followed by painful stimuli of the same intensity, pain ratings have in some studies been shown to be reduced (Babel et al., 2017), and this has been interpreted as a placebo analgesic response. However, in at least one other study (Carlino et al., 2015) pain ratings were not reduced when a conditioning only procedure was used, as in the present study. Even though these studies are methodologically different from many studies on placebo analgesia, where a sham treatment is administrated, they are conceptually similar. Together, these studies raise the important issue of the role of expectations in placebo analgesia. Several studies have observed that placebos or cues can reduce pain in the absence of expectations (de Jong et al., 1996; Babel et al., 2017; Rhudy et al., 2018) whereas the present study showed that even if a capsule was rated as effective in reducing pain, a placebo effect was not observed.

Fifthly, the present study used capsules as the placebo intervention, whereas many other studies that have induced pain to the arm have used a cream (de Jong et al., 1996; Rhudy et al., 2018). However, placebo effects have been induced by administration of capsules or tablets in previous studies (Flaten et al., 1999), also when pain has been administrated to the arm (Flaten et al., 2006; Chouchou et al., 2015). Thus, the use of capsules was most likely not the reason why a reliable placebo effect was not observed.

Sixthly, this is the first study on placebo analgesia that has used a cold thermode to induce pain. As noted, several studies, e.g., Charron et al. (2006) and Czerniak et al. (2016) have observed placebo analgesia using the cold pressor test. However, not all studies using cold pain have been successful, and Tang and Colagiuri (2013) did not observe placebo analgesic responses with the cold pressor test. Petersen et al. (2012) applied a cold thermo roll for 2 s a hyperalgesic area at the chest, but did not observe a significant placebo effect. The cold thermo roll is similar to a thermode, but as it was applied for only 2 s to the chest of patients with neuropathic pain, it is difficult to compare with the presents results. Moreover, Petersen et al. (2012) did not find placebo effects for heat pain either, that has been used successfully in many studies. Roelofs et al. (2000) did not observe a placebo effect in the electrically elicited nociceptive flexion reflex nor in pain report. Likewise, Rhudy et al. (2018) did not find a placebo response in the nociceptive flexion reflex, although they did find a placebo effect in pain report. Thus, the use of the cold pain thermode was most likely not the main reason for the lack of placebo effect in the present study, as pain to other types of stimuli have also not been modified by placebos.

There could be several reasons for the lack of placebo analgesia in the present study, as noted. Placebo analgesia is mainly a top-down process initiated in frontal regions associated with expectations of treatment effects, that in turn activate mid-brain areas involved in modulation of pain via descending pathways (Schafer et al., 2018). In effect, reduced activity in pain-responsive areas in the brain has often been observed (e.g., Wager et al., 2004). This chain of events was not completed in the present study, as a mediation analyses indicated that even though the capsules were rated as more effective by the Conditioned group, this did not translate into a placebo analgesic response, as there was no association between effectiveness ratings and placebo analgesia. Likewise, there was no association between reduced pain in the manipulation trials, and reduced pain in the post-test. Thus, neither the conscious evaluation of the capsules as effective in reducing pain, or the automatic formation of an association of the capsules with reduced pain, had any effect on pain in the post-test. More pairings of the capsules with reduced pain could have increased the placebo analgesic effect, as suggested by Colloca et al. (2010), as capsule effectiveness ratings increased from the first to the second manipulation trial.

## Conclusion

In conclusion, conditioned placebo responses were not reliably observed. The placebo capsules were rated as effective painkillers, but this did not translate into a placebo analgesic effect in the post-test. This could be due to, e.g., violation of response expectancies, too few conditioning trials, and differences in pain ratings in the pre-test that could be due to previous experience with painkillers.

## Author Contributions

All authors contributed in the planning and analysis of the results. EB and PL ran the study, together with two research nurses. All authors have contributed to the writing of the manuscript, and have accepted that it can be published in Frontiers.

## Conflict of Interest Statement

The authors declare that the research was conducted in the absence of any commercial or financial relationships that could be construed as a potential conflict of interest.
