# Supplementary material for: Failure to Find a Conditioned Placebo Analgesic Response
Source: Front Psychol. 2018 Jul 30;9:1198. doi: 10.3389/fpsyg.2018.01198 (PMC6077950; doi:10.3389/fpsyg.2018.01198)
Supplement: Supplementary file 1 [file Data_Sheet_1.docx]

SUPPLEMENTARY MATERIALS

The placebo analgesic response could be assessed in two ways: as the between-group differences in the pre-test and post-test, which is reported in the manuscript. In addition, placebo analgesia could be assessed as the difference in reported pain between the Conditioned group and the Pain control group in the manipulation trials. These groups received the same pain stimulus in all tests, and the only difference between the groups was that the Conditioned group received the capsules whereas the Pain control group did not. These data are included as supplementary materials.

There was no indication of a “placebo effect” in the manipulation trials, as the Group by Test interaction was not significant (F(2,78) = 0.51, p = 0.61), and neither was the interaction of Group by Participant sex by Test (F(2,78) = 1.92, p = 0.16). A significant main effect of Test (F(2,78) = 38.12, p = 0.00001) was due to reported pain decreasing from the -10^0^C in pre-test (M = 5.59, (0.37)) to the 0^0^C administrated in the first manipulation trial (4.12 (0.38)), with a further decrease to the second manipulation trial (3.72 (0.44)) where a temperature of +5^0^C was administrated.

The significant interaction of Participant sex by Time (F(1,38) = 6.26, p = 0.17) was due to an increase in pain in females from 45 seconds to one minute and 45 seconds after onset of the painful stimulation (means of 4.74 (0.59) and 5.01 (0.56), respectively), while pain decreased in males (means of 4.19 (0.69) and 3.97 (0.66), respectively).

Experimenter sex

As the experiment was run with two male and two female experimenters, who ran 31 and 30 participants, respectively, experimenter sex was included as a factor in the analyses of the capsule effectiveness ratings and the pain ratings. However, as there were a number of other differences between the male and female experimenters, experimenter sex was not included as a factor in the article. The most notable differences were that the two males were PhD students in psychology, whereas the two females were nurses. The males were also younger than the females. These differences made it difficult to sort out the causes of any effects of experimenter sex.

Capsule effectiveness ratings: The interaction of Group by Experimenter sex (F(1,32) = 5.77, p = 0.023) was due to higher ratings in the Conditioned group only when a male acted as experimenter (Conditioned group = 5.01 (0.62) and Capsule control group = 1.75 (0.74)). There was no difference between the groups when a female acted as experimenter (Conditioned group = 2.40 (0.73) and Capsule control group = 2.45 (0.67)). The main effect of Experimenter sex was not significant (F(1,32) = 1.92, p = 0.18) and no other interaction involving Experimenter sex was significant.

Pain ratings in the pre-test: the main effect of Experimenter sex was not significant (F(1,49) = 2,90, p = 0.095) and no interaction involving Experimenter sex was significant.

Placebo analgesia: the main effect of Experimenter sex (F(1,49) = 4.37, p = 0.042) was due to lower pain report when a male acted as the experimenter (4.78 (0.28)) compared to when a female was the experimenter (5.63, (0.29)). However, Experimenter sex had no effect on the placebo analgesic response.
